# Supplementary material for: Chromosome-Level Genome Assembly and Annotation of the Highly Heterozygous Phallus echinovolvatus Provide New Insights into Its Genetics
Source: J Fungi (Basel). 2025 Jan 15;11(1):62. doi: 10.3390/jof11010062 (PMC11766896; doi:10.3390/jof11010062)
Supplement: Supplementary file 1 [file jof-11-00062-s001.zip › Supplementary.pdf]

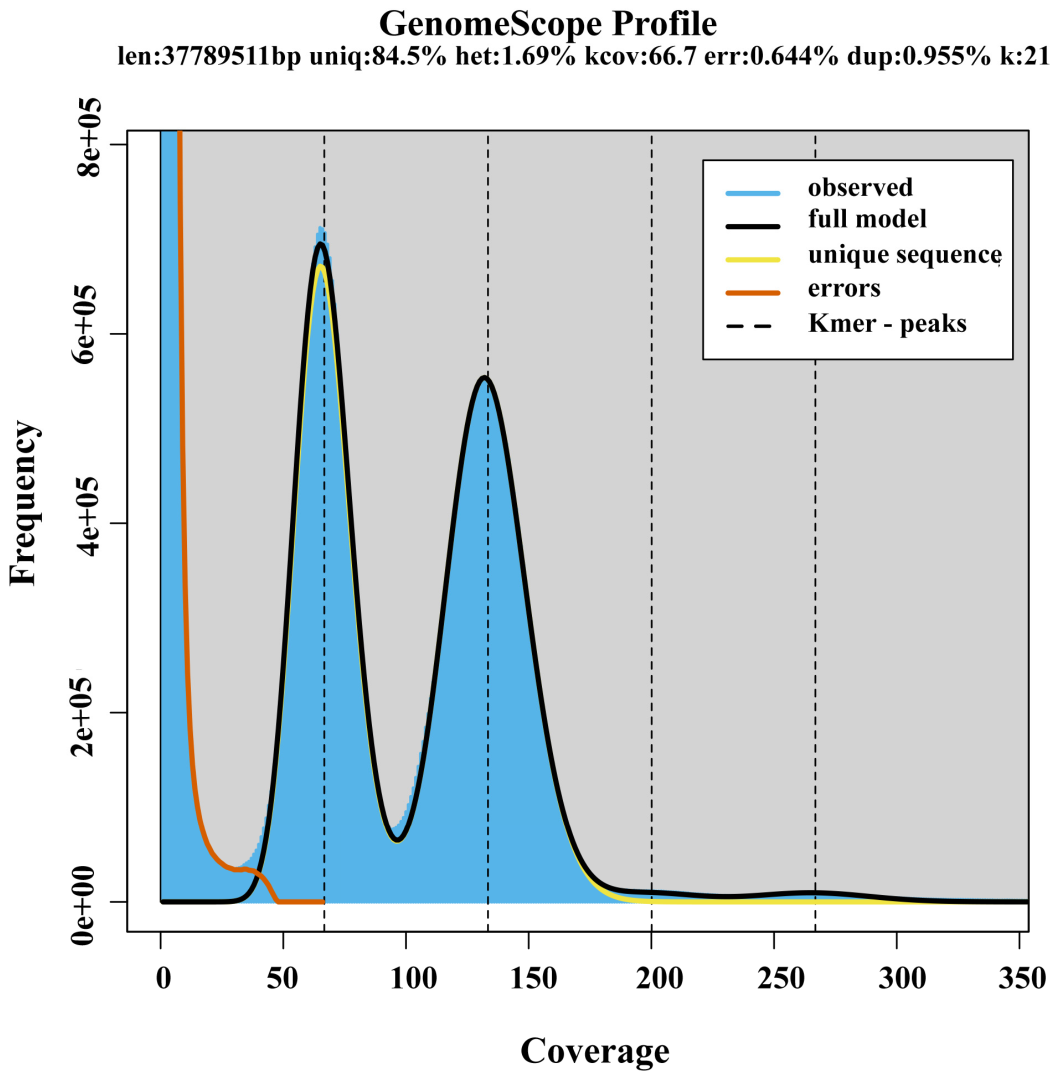

Figure S1. Histogram of the 21-mer depth distribution of the Illumina sequencing reads of *P. echinovolvatus*.

Blue areas indicate the observed k-mer frequencies and the black line indicates thefitted GenomeScope model. The blue line represents the actual k-mer curve, the black line is the k-mer curve estimated by the model, the yellow line is the k-mer curve corresponding to the unique data, the red line represents the error curve due to sequencing errors, and the dashed line represents speculation k-mer peak.

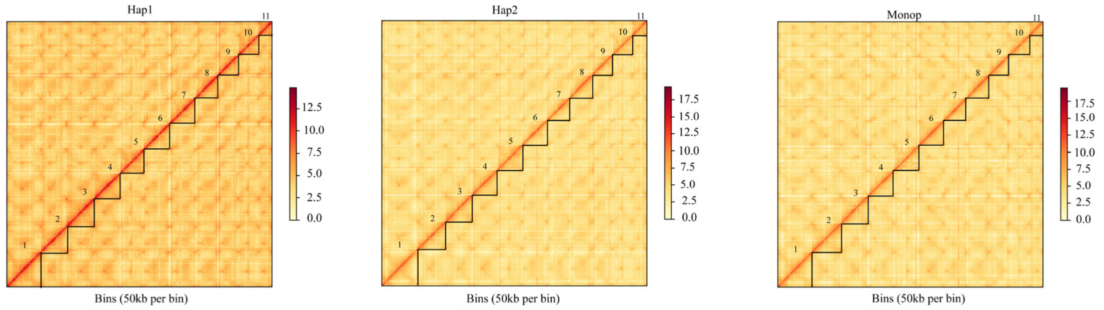

Figure S2. Whole-genome Hi-C interaction map at 50k resolution.

The triangular boxes represent the 11 chromosomes, with 1 to 11 indicating the chromosome numbers.

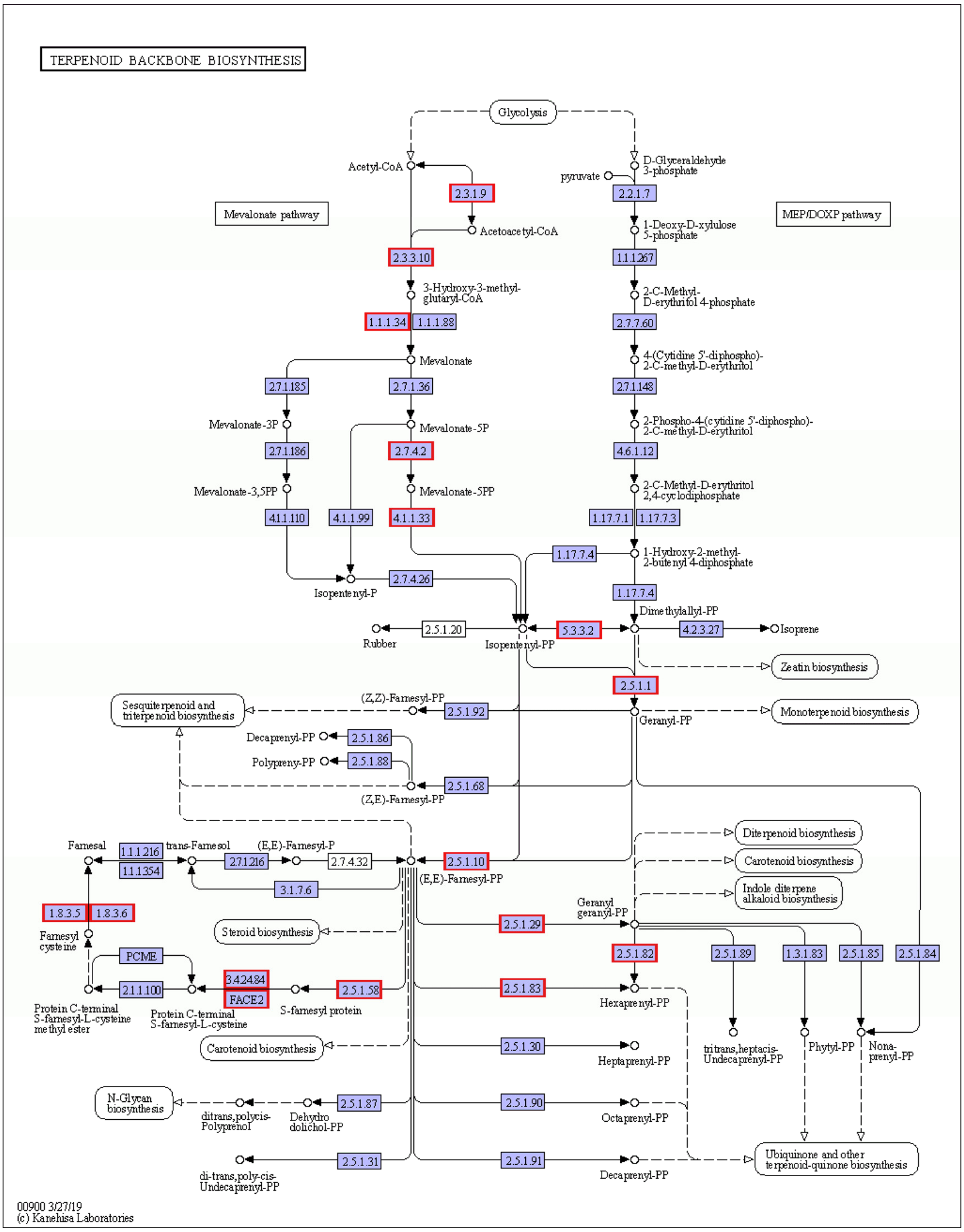

Figure S3. “Terpenoid backbone biosynthesis” pathway of *P. echinovolvatus*. The red box indicates existing homologous genes of the enzyme, while the white box means not.
